# Near-Infrared Light-Remote Localized Drug Delivery Systems Based on Zwitterionic Polymer Nanofibers for Combination Therapy

**DOI:** 10.3390/polym14091860

**Published:** 2022-05-01

**Authors:** Yu-Lun Li, Ching-Yi Chen

**Affiliations:** Department of Chemical Engineering, National Chung Cheng University, Chia-Yi County 621301, Taiwan; j98877661@gmail.com

**Keywords:** electrospun nanofibers, zwitterionic polymer, controlled release, indocyanine green, photothermal therapy

## Abstract

Localized drug delivery systems (LDDS) have gained great interests because they can directly treat the tumors and minimize systematic toxicity, and maximize drug action by controlling release precisely at the tumor site. However, the resistance of the non-specific adsorption of biomolecules is also important to alleviate the inflammatory reactions and avoid the decrease in performance of LDDS. In this study, we develop a near infrared (NIR) light-triggered nanofibrous delivery system consisting of zwitterionic poly(2-methacryloyloxyethyl phosphorylcholine)-*b*-poly(ε-caprolactone) (PMPC-*b*-PCL) encapsulated with indocyanine green (ICG) and doxorubicin (DOX) for dual photothermal therapy and chemotherapy. The nanofibrous mat shows hydrophilic characteristics and good antifouling performance. Under mild NIR irradiation, ICG could convert NIR light into thermal energy that elevates the surrounding temperature above 45 °C. This thermal energy also markedly accelerates the DOX release from the nanofibrous mat due to softening of the nanofibers, indicating the drug release could be controlled and switched on/off by light-triggering. Moreover, this light-triggered thermal energy and releasing behavior contribute to enhancing the cell lethality. Intracellular DOX distribution confirms the more drugs release upon light irradiation. All results demonstrate the developed light-triggered drug release nanofibers as LDDS are biocompatible and antifouling as well as has the superior combinational chemotherapy/photothermal therapy.

## 1. Introduction

In the past few decades, the delivery methods of drugs have attracted greater attention because they influence not only the therapeutic efficacy of a drug, but also the pharmacokinetics, pharmacodynamic, distribution, metabolism, and systemic toxicity [1]. Combined with the nanotechnologies, development of a new drug delivery system, such as micelles, liposomes, nanoparticles, nanogels, and nanofibers, emerges as a new promising and potential tool in the pharmaceutical field. These nano-sized delivery systems can carry toxic, insoluble, or unstable drugs, avoid rapid clearance by the human body, and provide passive or active specific targeting functions on the target site. Among these delivery systems, nanofibers have many desirable properties, such as high drug loading capacity, good encapsulation efficiency, unique physiochemical properties (e.g., large surface area and high porosity), offering localized delivery of drugs, simultaneous delivery of diverse therapies and cost-effectiveness [2,3,4]. Moreover, in situ application or implantation of the nanofibers can reduce systemic toxicity and the widespread distribution of free drugs in the body [5]. Especially used as implantable nanofibrous mats for solid cancer chemotherapy or after surgery, the local drug delivery in cancer therapy can avoid oral or systemic drug delivery that leads to inevitable adverse effects associated with chemotherapeutics. On the other hand, nanofibers introduced with external or internal stimuli-responsive functions have demonstrated tremendous potential for on-demand drug release in a dose-, spatial-, and temporal-controlled manner at the target site, which respond to intracellular biological stimuli (e.g., pH, redox potential, and enzyme) as well as external triggers (e.g., light, temperature, magnetic field and ultrasound) [6,7]. It has been reported to promote the therapeutic efficacy against tumors and minimize the toxicity on normal tissues [8,9].

Near infrared light (NIR) as a superior external trigger has gained a great interest in biomedical use because it is a noninvasive method, can be quickly switched on-off, easily be focused in specific areas, as well as can easily manipulate intensity and wavelength of the light source for precise control of drug release. In addition, it is a simple and feasible approach to achieve both spatial and temporal controlled drug release. Nakielski et al. reported PLLA nanofibers loaded with a model drug RhB, which encapsulates thermo-responsive P(NIPAAm-*co*-NIPMAAm) hydrogel containing gold nanorods (AuNRs) [10]. The drug release can be controlled by NIR light, which is absorbed by AuNR and converted into heat. The generated heat, in turn, causes structural changes of thermo-responsive hydrogel and accelerates the drug release rate. Huang and co-workers reported the development of poly(vinyl alcohol) (PVA) fibrous hybrids embedded with upconversion nanoparticles (UCNPs) and UV-cleavable levofloxacin conjugates for wound dressings. Upon UCNPs exposed to NIR light, they produce UV light that breaks the o-nitrobenzyl linkage of the levofloxacin conjugates in the PVA fiber, resulting in controlled drug release [11].

Electrospinning technique is a facile and low-cost method to fabricate nano- or micro-sized continuous fibers with uniform dispersion of drug within the polymeric matrix for drug delivery [3,12]. The versatile biodegradable, biocompatible, or stimuli-responsive polymers have been used to fabricate the nanofibers for drug delivery system [13]. For in situ applications and medical implants, the antifouling coatings are a considerable demand that can resist nonspecific protein adsorption and cell attachment to avoid reducing their performance efficiency and causing thrombosis-like side effects and foreign body reactions [14,15]. Zwitterionic polymers are a new generation of antifouling materials, which consist of both positively and negatively charged moieties located on the same repeating unit. The strong surface hydration produced from the tightly bound water layer via electrostatic attractions acts as a physical barrier to prevent the adhesion of proteins and microbe. Their strong electrostatic interaction with water molecule exhibits the outstanding antifouling properties compared to the hydrogen bonding employed by conventional antifouling materials, poly(ethylene glycol) (PEG), or poly(2-hydroxyethyl methacrylate) (PHEMA). Moreover, the oxidation problem of PEG causes it to lose hydrophilicity, which is not suitable for long-term applications, while PHEMA still induces foreign body response due to its partially nonspecific protein adsorption resistance [14]. Therefore, zwitterionic polymers are expected to be a promising alternative to conventional antifouling materials. Among zwitterionic polymers, poly(2-methacryloyloxyethyl phosphorylcholine) (PMPC) containing phosphorylcholine moiety that is the major component in the outer membrane of most cell has been demonstrated to reduce thrombogenic effects and biofouling [14,16,17]. This unique antibiofouling property can remarkably minimize subsequent adverse inflammatory reactions and prevent the decrease in performance of biomedical applications, such as medical implants, drug delivery carriers etc. Herein, in this study, we develop a NIR light-triggered nanofibrous delivery system that can repeatedly generate heat for photothermal therapy and simultaneously trigger the release of encapsulated anticancer drugs. This nanofiber membrane consists of poly(2-methacryloyloxyethyl phosphoryl-choline)-*b*-poly(ε-caprolactone) (PMPC-*b*-PCL) loaded with both photothermal agent indocyanine green (ICG) and anticancer drug doxorubicin (DOX). The zwitterionic PMPC segment can increase the hydrophilicity and provide prominent ultralow biofouling capability of nanofibers, which is expected to reduce non-specific protein adsorption on the membrane. The PCL segment has good biocompatibility and biodegradability, and adequate mechanical properties [18]. We expect the ICG can absorb NIR irradiation to convert into heat not only leading to the thermal ablation of cancer cells, but also triggering DOX release from the nanofibers due to the melting of the nanofibers. The purpose is to develop an antifouling localized delivery system to achieve NIR remote drug release and a combination effect of chemotherapy and photothermal therapy (PTT) in treating cancer cells.

## 2. Materials and Methods

### 2.1. Materials

2,2′-bipyridine (bpy, 98%, Alfa Aesar, Tewksbury, MA, USA), bovine serum albumin-conjugated FITC (BSA-FITC, Thermo Fisher Scientific, Waltham, MA, USA), 4′, 6-diamidino-2-phenylindole (DAPI, Thermo Fisher Scientific, Waltham, MA, USA), indocyanine green (ICG, TCI, Tokyo, Japan), 2-methacryloyloxyethyl phosphorylcholine (MPC, Sigma Aldrich, St. Louis, MO, USA), tetrabutylammonium iodide (TBAI, Acros, Fair Lawn, NJ, USA) were used as received. Copper bromide (CuBr, >99.998%, Alfa Aesar, Tewksbury, MA, USA) was washed with acetic acid, diethyl ether, and then dried under vacuum. ε-caprolactone was distilled over CaH_2_ under reduced pressure. Triethylamine (TEA, J.T. Baker, Phillipsburg, NJ, USA) were distilled under N_2_. All solvent were purchased from Sigma-Aldrich, ECHO, or Macron and used as received unless otherwise noted. Doxorubicin hydrochloride (DOX HCl) was purchased from Seedchem Company (Melbourne, Australia). Bicinchoninic acid (BCA) protein assay kit (Thermo Fisher Scientific, Waltham, MA, USA) was performed according to manufacturer′s instruction. The (3-(4,5-dimethyl thiazol-2-yl)-2,5-diphenyl-tetrazolium bromide) (MTT) assay was obtained from Bio Basic (Markham, ON, Canada). Minimum essential medium (MEM 1X) and trypsin EDTA (1X) were purchased from Corning (Corning, NY, USA). Phosphate-buffered saline (PBS, 1X), amino acid (100X), sodium pyruvate (100 mM), and penicillin-streptomycin solution (100X) were commercially available from BioWest (Nuaillé, France). Ultrapure water was used in all experiments. Hydroxyethyl 2-bromoisobutyrate (HEBiB) and poly(ε-caprolactone) macroinitiator (PCL-Br) were prepared according to our published protocols [19].

### 2.2. Synthesis of PCL-b-PMPC (P1 and P2)

PCL-*b*-PMPC was synthesized by atom transfer radical polymerization [19,20] using PCL-Br as macroinitiator with feeding molar ratios of MPC monomer to PCL-Br macroinitiator fixed at 60/1 for **P1** and 100/1 for **P2**, respectively. Typically for synthesis of **P1**, PCL-Br macroinitiator (1.5 g, 0.039 mmol, DP_PCL_ = 334, M_n H-NMR_ = 38,100 g/mol, M_nGPC_ = 26,400, M_w_/M_n_ = 1.9) and CuBr (17 mg, 0.17 mmol) were added into a Schlenk tube and then vacuumed for 30 min. A solution of MPC (0.7 g, 2.37 mmol) and bpy ligand (12 mg, 0.076 mmol) in 5.5 mL DMSO and 2.5 mL MeOH was degassed by purging with nitrogen for 30 min and then transferred into the Schlenk tube followed by freezing–pumping–thawing three times. After stirring at ambient temperature for 20 min, the mixture was polymerized at 85 °C under nitrogen for 16 h. The resulting reaction solution were purified by dialysis using dialysis membrane (MWCO 12,000-14,000 Da, Cellu-Sep, Interchim Inc., Los Angeles, CA, USA) against a MeOH/THF (1:1, *v*/*v*) solvent mixture for 48 h and the solvent mixture was replaced every 6 h. Finally, the product was precipitated into cold diethyl ether and dried to obtain white powder **P1** (1.5 g). **P1**: DP_MPC_ = 30, M_n H-NMR_ = 46,900 g/mol. **P2**: DP_MPC_ = 63, M_n H-NMR_ = 56,700 g/mol. The ^1^H-NMR spectra of purified **P1** and **P2** was recorded using a CDCl_3_/CD_3_OD (1:1, *v*/*v*) mixture (Figure S1, Supplementary Materials).

### 2.3. Preparation of Electrospun Nanofiber Membranes

PCL-*b*-PMPC (**P1** or **P2**) was dissolved in MeOH/CHCl_3_ (1:2, *v*/*v*) with polymer concentration of 16 wt% and the mixture was stirred for 4 h at room temperature. The ES nanofiber membranes (ENMs) were prepared using a single-capillary spinneret. The polymer solution was fed into a 22 gauge metallic needle using syringe pump (Fusion 100, Chemyx Inc., Stafford, TX, USA) at a flow rate of 0.3 mL/h. The metallic needle was connected to a high-voltage power supply (You-Shang Technical Corp., Kaohsiung, Taiwan) set at 15 kV, and a piece of aluminum foil was placed 15 cm below the tip of the needle to collect the nanofibers for 2 h. For comparison, PCL nanofibers were prepared at a higher polymer concentration of 25 wt% under the same electrospinning conditions.

Before preparation of drug-loaded nanofibers, DOX HCl in DMSO (100 mg/mL) was neutralized to free base by adding 6 eq. of triethylamine and stirring in dark overnight, and ICG was pretreated with 6 eq. of TBAI in DMSO to form a hydrophobic ICG-tetrabutylamine salt as reported in previous literature [21]. Then, 4% of DOX and/or 8% of ICG (*w*/*w* of total **P2** polymer) were added to polymer solution with concentration of 16 wt% and stirred for 4 h in dark. The applied voltage was set at 22 kV with feed rate of 0.15 mL/h. Both ICG- and DOX-loaded nanofibers are designated as **P2ID**, and only ICG- or DOX-loaded nanofibers are designated as **P2I** or **P2D**, respectively. The thickness of the nanofiber membrane was 0.043 mm. All experiments were carried out at room temperature and around 30% relative humidity.

The prepared nanofibers were weighted and then washed with PBS (pH 7.4) solution to remove the excessive ICG and DOX attached. The drug-loaded content was determined using UV–visible spectra monitoring the absorbance at 799 nm with reference to a calibration curve of ICG (Figure S2a, Supplementary Materials) and at 500 nm with reference to a calibration curve of DOX in MeOH/CHCl_3_ (1:2, *v*/*v*) mixture (Figure S2b, Supplementary Materials). The drug loading content (DLC) was calculated from the weight ratio of drug loaded in nanofibers to total weight of nanofibers. All drug-loading experiments were repeated three times.

### 2.4. General Characterization

^1^H NMR spectra were measured in CDCl_3_ and methanol-d_4_ by using a Bruker-DPX-400 instrument spectrometer (Bruker, Rheinstetten, Germany) operating at 400 MHz. Molecular weights and polydispersity of PCL-Br were determined using waters gel permeation chromatography (GPC, Waters, Milford, MA, USA) coupled with refractive index detectors (Waters 2410), in reference of a series of polystyrene standards (absolute molecular weight from 980 to 2,110,000 g/mol) with THF as the eluent (1 mg/mL) at 40 °C. The morphologies of the ES nanofibers were characterized by field-emission scanning electron microscopy (FE-SEM, Hitachi S-4800, Tokyo, Japan). Before imaging, the samples were sputtered with Pt. The average diameter of nanofibers was estimated over fifty fibers in SEM images from each sample. Static water contact angles were measured using a contact angle goniometer (CAM-100, Creating Nano Technologies Inc., Tainan, Taiwan). A water droplet (about 5 μL) was deposited on the nanofibrous membrane fixed onto a glass slide. Photographs of water/nanofiber interface was recorded. The absorption spectra were measured with UV–vis spectrometer (JASCO V-570, Jasco, Tokyo, Japan). The photoluminescence spectra (PL) were recorded with a Hitachi F-4500 spectrophotometer (Hitachi, Tokyo, Japan).

### 2.5. Quantitative and Qualitative Evaluation of Protein Adsorption

**P1** and **P2** nanofiber membranes of 1 × 1 cm^2^ dimensions were first rinsed with PBS buffer and immersed into 1 mL of freshly prepared bovine serum albumin (BSA) or BSA-FITC solution (1.5 mg/mL) at 37 °C for 4 h. The membranes were then washed thoroughly by PBS three times. For quantitative evaluation of protein adsorption [22], the resulting nanofibers were placed into 1 mL of 1 wt% SDS solution and ultrasonicated for 20 min to detach the adsorbed protein on the surface. BCA working reagent prepared according to manufacturing protocol was added to the protein sample with a sample to working reagent ratio of 1:8 (*v*/*v*), followed by incubated at 37 °C for 30 min. The concentration of BSA protein was determined by measuring the absorbance at 562 nm using a microplate reader (BioTek-MQX200, Agilent, Santa Clara, CA, USA). For qualitative assessment of protein adsorption on the surface of nanofibers, the BSA-FITC protein adsorbed nanofibers was observed under a confocal laser scanning microscope (CLSM, Olympus FV 1000, Olympus, Tokyo, Japan). PCL nanofiber membrane was used as a control group.

### 2.6. Photothermal Effect

To evaluate the heat generation from nanofibers by irradiation with 808 nm laser at a power density of 0.78 W/cm^2^, 1 × 1 cm^2^ **P2ID** nanofibers (containing ICG 30 μM) were immersed in PBS (pH 7.4, 6 mL) and irradiated for 20 min. A thermocouple (PTM-806, Lutron, Coopersburg, PA, USA) was placed into the solution to record the solution temperature in 1 min intervals. Free ICG (30 μM) and **P2** nanofibers were prepared and monitored with the same procedure as control groups. For photothermal stability study, **P2ID** nanofibers (5 mm in diameter, containing ICG 100 μM) were immersed in PBS (pH 7.4, 200 μL) and then the solution was exposed to NIR light at a power density of 0.78 W/cm^2^ for 3 min, followed by natural cooling to room temperature without NIR irradiation for 3 min. The temperature change of solution was recorded by a thermocouple over five on/off cycles.

### 2.7. Drug Release Behavior

**P2ID** and **P2D** nanofiber membranes were fixed onto glass substrate and immersed in 6 mL PBS (pH 7.4) solution. The membranes were irradiated with an 808 nm NIR laser at a power density of 0.78 W/cm^2^ for 10 min and the diameter of light spot around 1 cm could nearly cover the nanofibers. Then, the light source was turn off for 2 h. This process was repeated several times to investigate the light-remote release behavior. At predetermined time intervals, 6 mL of buffer solution was collected and replaced with an equal volume of fresh PBS. The collected solution was lyophilized and redissolved in MeOH/CHCl_3_ (1:2, *v*/*v*) mixture. The amount of DOX released was determined by absorbance measurements. The nanofibers without NIR irradiation were used as control groups.

### 2.8. Cell Culture and In Vitro Cytotoxicity and Phototoxicity

Human cervical cancer cell line (HeLa cells) was cultured in Minima essential medium (MEM) supplemented with 10% (*v*/*v*) fetal bovine serum (FBS), 1% (*v*/*v*) penicillin-streptomycin, sodium pyruvate, and amino acid and then incubated in a humidified atmosphere containing 5% CO_2_ at 37 °C. HeLa were seeded in 48-well plates at a density of 3 × 10^4^ cells per well and incubated at 37 °C in 5% CO_2_ atmosphere for 18 h. Then different nanofiber membranes of 1.5 × 1.5 cm^2^ dimension were first rinsed with PBS and carefully placed into the wells of each plate by adding 500 μL of fresh medium. Unloaded **P2**, singe drug-loaded **P2I** and **P2D**, and dual drugs-loaded **P2ID** nanofiber membranes were used in this study. The drug-loaded nanofibers contained 80 μg of DOX and/or 83 μg of ICG. Each sample was conducted in three replicates per plate. The synergistic effect of PTT and chemotherapy was conducted in the absence and presence of an 808 nm NIR laser (0.47 W/cm^2^) irradiation for 5 or 10 min. After that, the plates were incubated for another 24 h. The cell viability was evaluated with MTT assay. The data are given as mean ± standard deviation (SD).

### 2.9. Subcellular Localization Study

To evaluate the photothermal drug release behavior, HeLa cells were grown on coverslips in 24-well plate at a density of 1 × 10^6^ cells per well for 24 h. Then, **P2ID** membrane of 1.5 × 1.5 cm^2^ dimension was placed into the well by adding 800 μL of fresh medium. The corresponding well was irradiated for 10 min (808 nm, 0.47 W/cm^2^) and further incubated for 24 h in a humidified atmosphere containing 5% CO_2_ at 37 °C. After removal of membrane and rinsing with PBS, the cells were fixed in 4% paraformaldehyde for 10 min, followed by staining with DAPI (10 μg/mL) for 10 min and mounted on a slide. The stained samples were examined using CLSM.

## 3. Results and Discussion

### 3.1. Preparation and Characterization of PCL-b-PMPC Copolymers and Electrospun Nanofibers

In this study, a series of biocompatible and zwitterionic-based block copolymer poly(ε-caprolactone)-*b*-poly[(2-methacryloyloxy)ethyl phosphorylcholine] (PCL-*b*-PMPC, **P1** and **P2**) was synthesized via ring-opening polymerization (ROP) and atom transfer radical polymerization [19] as depicted in Scheme S1 (in the Supplementary Materials). The PCL block with alkyl bromide terminated was prepared according to our previous synthetic procedure [23] and used as macroinitiator to polymerize MPC segment with various MPC/PCL-Br molar ratios under ATRP condition. **P1** has the shorter PMPC segment, whereas **P2** has the longer PMPC length. We varied the length of the MPC segment to investigate its hydrophilicity and resistance to protein adsorption. The purity and compositions of synthesized PCL-*b*-PMPC were characterized by ^1^H NMR spectra (Figure S1, Supplementary Materials).

**Figure 1:**
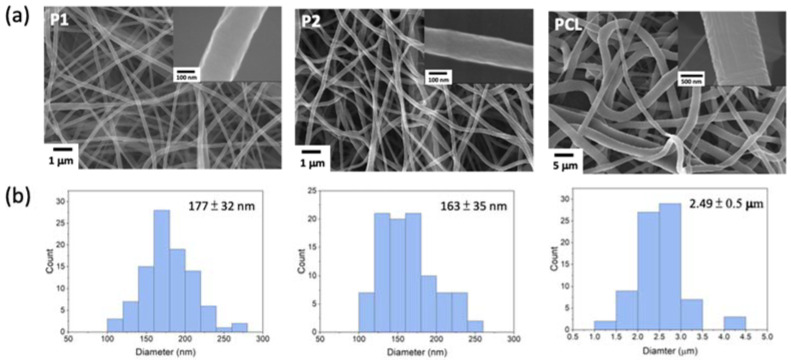
(**a**) FE-SEM images of the prepared ES nanofibers. The inset figures show the enlarged FE-SEM of ES nanofibers. (**b**) The corresponding size distributions of nanofibers: **P1**, **P2,** and PCL.

**Figure 2:**
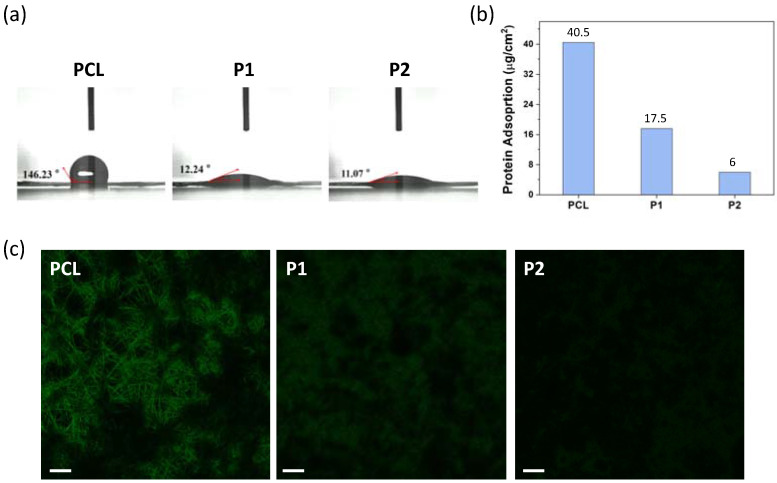
(**a**) Water contact angle images of the **PCL**, **P1** and **P2** nanofibers. (**b**) Adsorption of BSA proteins on the surfaces of different nanofibers. (**c**) CLSM images of BSA-FITC adhered on different nanofibers (scale bar is 10 μm).

Plain nanofibers of PCL-*b*-PMPC (**P1** and **P2**) and PCL were fabricated by electrospinning (ES) with polymer concentration of 16 wt% for PCL-*b*-PMPC and 25 wt% for PCL in methanol/chloroform solvent mixture, respectively. Figure 1 shows the FE-SEM images of both PCL-*b*-PMPC and PCL formed continuously and bead-free nanofibers with average diameters of 177 ± 32 nm for **P1**, 163 ± 35 nm for **P2**, and 2.49 ± 0.5 μm for PCL. The insert SEM images show a smooth and nonporous surface of all nanofibers. Compared to PCL for fabrication of nanofibers, PCL-*b*-PMPC required lower solution concentration and had smaller fiber diameters. This might be due to the presence of electrostatic interaction between zwitterionic groups and increase in the electrical conductivity of the polyelectrolyte solution that enabled the formation of uniform and thinner fibers [24]. The surface hydrophilic characteristics of the PCL-*b*-PMPC and PCL nanofibers were conducted by water contact angle measurement (Figure 2a). PCL nanofibers exhibited hydrophobic property with water contact angle of approximately 146°, while the electrostatic interaction with surrounding water molecules induced by zwitterionic structure of PMPC [25] caused **P1** and **P2** nanofibers with high wettability and minimal contact angle of 12° and 11°, respectively. This electrostatically induced hydration generated near the surface provides a physical and energetic barrier to prevent nonspecific protein adsorption and cell adhesion [25,26].

To investigate the antifouling property of PCL-*b*-PMPC nanofibers, bovine serum albumin (BSA), which can easily and strongly adsorb on a wide range of material surface, was used as the model protein. In this study, the adsorbed BSA protein was quantified by using the BCA assay and qualitatively evaluated by CLSM. As shown in Figure 2b, the hydrophobic PCL nanofibers showed high protein adsorption behavior (40.5 μg/cm^2^). Belfort et al. indicate non-polar surfaces destabilize proteins to change their conformations, resulting in strong protein–surface interaction [27]. However, the amount of adsorbed BSA was remarkably decreased with increasing PMPC content (17.5 μg/cm^2^ for **P1** and 6 μg/cm^2^ for **P2**), indicating incorporation of PMPC moiety can effectively improve the protein resistance by increasing hydration capacity and simulating cell membrane structure [14,16,17]. To qualitatively investigate the protein adsorption, nanofibers were contacted with BSA conjugated with FITC for 4 h (Figure 2c). It is obviously observed that the PCL-*b*-PMPC nanofibers exhibited very little protein adsorption in comparison with the PCL nanofibers. The extent of protein resistance was increased with PMPC content. Therefore, **P2** nanofibers with excellent antifouling property was chosen to encapsulate drugs for further study.

### 3.2. Drugs Encapsulation in Electrospun Nanofibers

**Figure 3:**
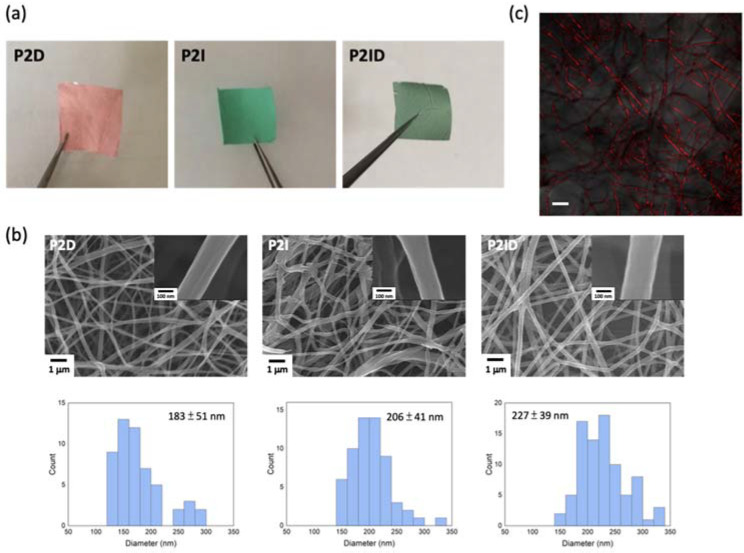
(**a**) Photographs of the **P2D**, **P2I**, and **P2ID** nanofibers. (**b**) FE-SEM images of the ICG and/or DOX loaded ES nanofibers. (**c**) Confocal images of **P2ID** nanofibers (scale bar is 2 μm).

Drug-loaded nanofibers were prepared by electrospinning of DOX- and/or ICG-added polymer solution. **P2ID** represents both ICG- and DOX-loaded nanofibers, and **P2D** and **P2I** represent the DOX-loaded and ICG-loaded nanofibers, respectively. The photographs of the **P2D**, **P2I**, and **P2ID** nanofibers are shown in Figure 3a. Figure 3b shows the FE-SEM images of the drug-loaded nanofibers having average diameters of 227 ± 39 nm (**P2ID**), 206 ± 41 nm (**P2I**), and 183 ± 51 nm (**P2D**). It can be observed that fibrous morphology was unaffected by the drug-loaded, but the fiber diameters were increased. The existence of both DOX and ICG in **P2ID** nanofibers was confirmed by UV-visible spectrum (Figure S3) and fluorescence image (Figure 3c). The confocal microscope images of **P2ID** nanofibers showed DOX fluorescent emission superimposed on nanofibers, indicating uniform distribution of DOX in the nanofiber structure. The loading content of DOX and ICG in the **P2ID** fibers was 27 and 27.5 μg/mg (drug weight per fiber weight), respectively.

### 3.3. Photothermal Effect under NIR Irradiation

**Figure 4:**
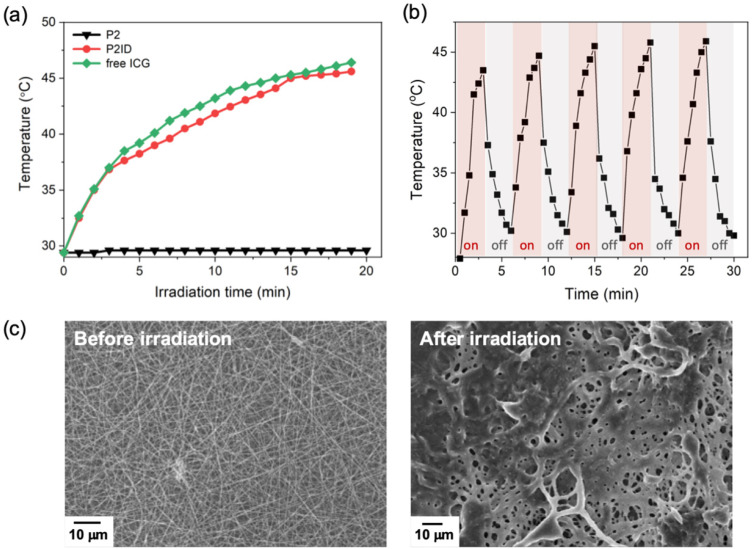
(**a**) Temperature profiles of **P2**, **P2ID** nanofibers and free ICG as a function of irradiation time under continuous NIR laser irradiation (a power density of 0.78 W/cm^2^). (**b**) Temperature change of **P2ID** nanofibers over five on/off cycles of repeated NIR laser irradiation (a power density of 0.78 W/cm^2^). (**c**) FE-SEM images of **P2ID** nanofibers before and after NIR irradiation at a power density of 0.78 W/cm^2^ for 10 min.

In this study, ICG was used not only to convert the NIR light energy into heat for photothermal therapy [28,29,30] but also to generate heat to soften nanofibers for accelerating DOX release. The heating behaviors of **P2ID** nanofibers under NIR light at different time intervals were monitored by the temperature increment in aqueous solution. As shown in Figure 4a, negligible temperature change was found in **P2** nanofibers, which was in the absence of ICG. However, in the presence of ICG, NIR light gradually elevated the solution temperature of **P2ID** nanofibers to 45.5 °C with the same photothermal effect as free ICG to 46.4 °C. This indicated the ICG in nanofibers could provide a significant photothermal effect and the temperature above 43 °C was sufficient to induce an irreversible damage to cancer cell [29,31]. Figure 4b shows the photothermal stability of **P2ID** nanofibers after five on/off cycles of NIR irradiation. The temperature elevations of **P2ID** nanofibers exhibited no visible decrease and the temperature change was reproducible during five cycles, indicating the **P2ID** nanofibers have excellent photostability. The softened fibrous structures of **P2ID** nanofibers after NIR irradiation were observed by FE-SEM (Figure 4c), confirming the heat generation and phase transition of **P2ID** nanofibers can occur remotely by NIR light. This light triggered characteristic can also be used as a switch to accelerate the drug release from nanofibers.

### 3.4. In Vitro NIR Light Remote Release Behavior

**Figure 5:**
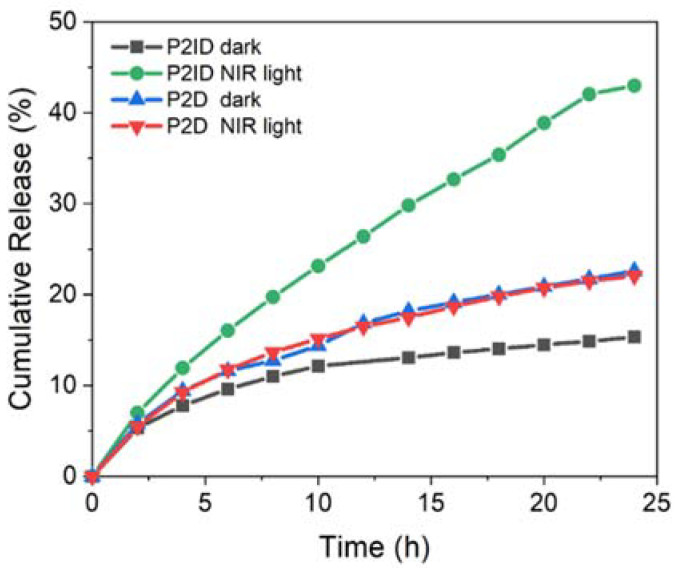
Cumulative DOX release from **P2ID** and **P2D** nanofibers in the absence and presence of NIR irradiation (0.78 W/cm^2^).

The NIR light-dependent release behaviors of **P2ID** nanofibers were evaluated by intermittently irradiating the fibers for 10 min every 2 h and monitored the DOX release using absorbance measurements (Figure 5). The **P2ID** fibers without NIR irradiation and **P2D** fibers in the absence of ICG with and without irradiation were evaluated at the same procedure as the control groups. Upon NIR irradiation, ICG absorbed the NIR light energy and converted it into heat that resulted in a faster release rate of DOX from nanofibers with about 43% after 24 h. The increase of temperature within the fibers induced by NIR irradiation softened the nanofibers and facilitated the DOX release. In the control experiments, the cumulative release amount was only 15% for **P2ID** fibers without NIR irradiation and 20% for **P2D** fibers regardless of irradiation after 24 h. This is much lower than the NIR-triggered **P2ID** nanofibers. The minor DOX release was due to the high hydrophobicity and slow hydrolysis mechanism of the PCL segment, which suppressed the drug diffusion. The slightly faster DOX release rate from **P2D** fibers than that of **P2ID** fibers without irradiation was possibly due to the thinner fiber diameters of **P2D** (183 ± 51 nm) than that of **P2ID** fibers (227 ± 39 nm). These results demonstrated the drug release process could be successfully manipulated by ICG within nanofibers via photothermal effect. This NIR light remote system can achieve precisely and actively controlled drug release by irradiation.

### 3.5. In Vitro Biocompatibility, Anticancer Efficiency, and Cellular Uptake of Released DOX

**Figure 6:**
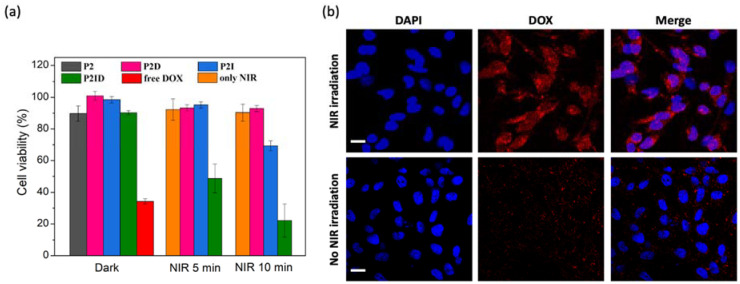
(**a**) Cell viability of HeLa cells treated with various nanofibers and free DOX in the absence and presence of NIR irradiation (0.47 W/cm^2^). (**b**) CLSM images of HeLa cells treated with **P2ID** nanofibers with or without NIR irradiation (0.47 W/cm^2^, 10 min). The scale bar is 20 μm.

Good biocompatibility is an essential factor for biomedical applications. Thus, the cytotoxicity of plain **PCL** and **P2** nanofibers were investigated against L929 cells. Cells seeded on culture plate without any treatment acted as the control group. As shown in Figure S4, both the **PCL** and **P2** nanofibers had good L929 cell compatibility with no cytotoxicity, which was suitable for biomedical applications. In order to evaluate the combined therapeutic effect of **P2ID** nanofibers for cancer cells under NIR light irradiation, in vitro cytotoxicity of free DOX, **P2ID**, **P2D,** and **P2I** nanofibers with or without 808 nm laser irradiation were examined using HeLa cells via MTT assay. The drug-loaded nanofibers contained 27 μg/mg of DOX and/or 27.5 μg/mg of ICG. As shown in Figure 6a, without laser irradiation, the cell viability of **P2** nanofibers incubated with HeLa cells for 24 h was around 90%, indicating good biocompatibility and no significant cytotoxicity of **P2** nanofibers. The cell viability of drug-loaded nanofibers (**P2ID**, **P2D**, **P2I**) in the dark also showed high cell survival due to the less amount of drug release. Before confirming the anticancer efficacy of **P2ID** nanofibers for the application of combination therapy, the cells treated with NIR laser light at a power density of 0.47 W/cm^2^ for 5 or 10 min showed no phototoxicity in our experimental conditions. Then, **P2ID** nanofibers were used to evaluate the combined chemotherapy and photothermal therapeutic effect. The results showed the viability of HeLa cells had an irradiation-dependent nature, from 90.3% to 51.8% and 22.2% for 5 and 10 min irradiation, respectively. The significantly enhanced cell lethality can be attributed to the fast release of DOX triggered by photothermal heating resulted from ICG under irradiation. Moreover, ICG converted light energy into heat under NIR irradiation, which could provide hyperthermia effect on killing cancer cell. On the other hand, the cells cultured with **P2D** nanofibers under irradiation showed no apparent cell death, indicating the slow release of DOX in the absence of ICG even under NIR irradiation. For **P2I** nanofibers, the cell viability was about 95.2% and 69.3% after 5 and 10 min laser irradiation, respectively. The less cytotoxicity of **P2I** fibers compared to that of **P2ID** fibers suggested only the hyperthermia effect from ICG had low therapeutic effect on killing cells; however, combinational chemotherapy/photothermal therapy had dramatically enhanced the therapeutic efficiency.

Confocal laser scanning microscopy (CLSM) was used to investigate the subcellular localization of the released DOX from **P2ID** nanofibers in response to light irradiation in HeLa cells. A blue-fluorescent dye DAPI was used to stain the cell nuclei. HeLa cells treated with **P2ID** nanofibers in the absence of NIR light irradiation showed less DOX fluorescence existence in the cytoplasm and cell nuclei after 20 h incubation (Figure 6b). However, **P2ID** nanofibers with NIR irradiation displayed markedly enhanced fluorescent signals of DOX and collocated in the cell nuclei. The results demonstrated the NIR light can remotely trigger the release of DOX from **P2ID** nanofibers. All results evidently demonstrated the **P2ID** nanofibers were biocompatible and had superior combination therapeutic efficiency.

## 4. Conclusions

In this work, we prepared a NIR light-triggered localized drug delivery system (LDDS) based on zwitterionic PCL-*b*-PMAC copolymer, electrospinning technique, and dual indocyanine green (ICG) and doxorubicin (DOX) encapsulation for combination therapy. This light-triggered LDDS system (**P2ID** nanofibrous mat) can effectively convert light to thermal energy and simultaneously accelerate the release of encapsulated DOX when activated by NIR light. The developed **P2ID** mat also exhibits excellent antifouling property and biocompatibility. Intracellular DOX distribution confirms more drug release due to the light triggered release. Moreover, the **P2ID** mat can generate heat and release anticancer drugs, thus causing significant death of cancer cells. We demonstrate that the light-triggered **P2ID** LDDS presents excellent antifouling property, superior combinational chemotherapy/photothermal therapy on cell growth inhibition, and an effective treatment with low systemic toxicity for treating cancers.

## Data Availability

Not applicable.

## Supplementary Materials

The following are available online at https://www.mdpi.com/article/10.3390/polym14091860/s1. Scheme S1: Synthetic route for (**a**) PCL-Br and (**b**) PCL-*b*-PMPC copolymers, Figure S1: ^1^H-NMR spectrum of (**a**) PCL-Br in CDCl_3_ and (**b**) PCL-*b*-PMPC copolymers in CDCl_3_/CD_3_OD (*v*/*v*, 1:1), Figure S2: The calibration curves of (**a**) ICG and (**b**) DOX in MeOH/CHCl_3_ (1:2, *v*/*v*) mixture, Figure S3: UV-vis spectrum of free ICG and free DOX (in solution, CHCl_3_/MeOH, 2/1, *v*/*v*), and both DOX and ICG-loaded nanofibers (P2ID), Figure S4: Cell viability of L929 cells treated with plain PCL and P2 nanofibers.
