# Supplementary material for: Near-Infrared Light-Remote Localized Drug Delivery Systems Based on Zwitterionic Polymer Nanofibers for Combination Therapy
Source: Polymers (Basel). 2022 May 1;14(9):1860. doi: 10.3390/polym14091860 (PMC9100176; doi:10.3390/polym14091860)
Supplement: Supplementary file 1 [file polymers-14-01860-s001.zip › polymers-1696977-supplementary.pdf]

Electronic supplementary material for:

Near-Infrared Light-Remote Localized Drug Delivery Systems Based on Zwitterionic Polymer Nanofibers for Combination Therapy

Yu-Lun Li, Ching-Yi Chen*

Department of Chemical Engineering, National Chung Cheng University, Chia-Yi County, 62102, Taiwan

Correspondence to: C.-Y. Chen (E-mail: *chmcyc@ccu.edu.tw*)

In vitro biocompatibility. L929 cells were seeded in 24-well plates at a density of 5 × 10^4 cells per well and incubated at 37 ºC in 5% CO_2 atmosphere for 24 h. Then, nanofibers cut into discs (5 mm in diameter) were first sterilized with 75% ethanol, followed by rinsing with PBS and carefully placed into the wells of each plate with adding 2 mL of fresh medium. The culture plate was incubated for 24 h. After that, the nanofiber discs and medium were removed and MTT assay was used to evaluate the cell viability. Each sample was conducted in three replicates.

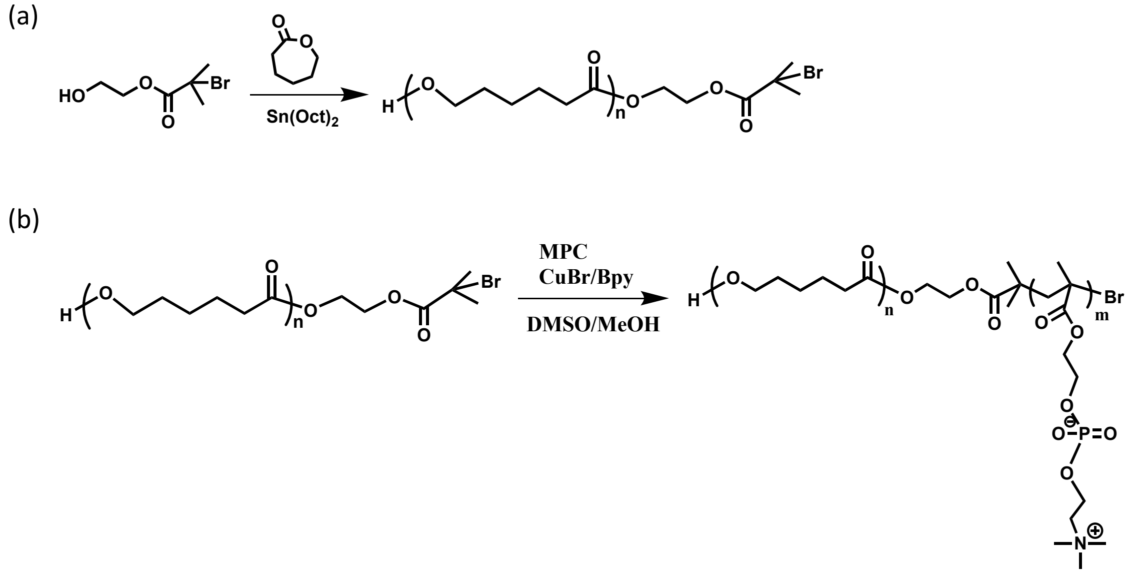

Scheme S1 Synthetic routes for (a) PCL-Br and (b) PCL-*b*-PMPC copolymers

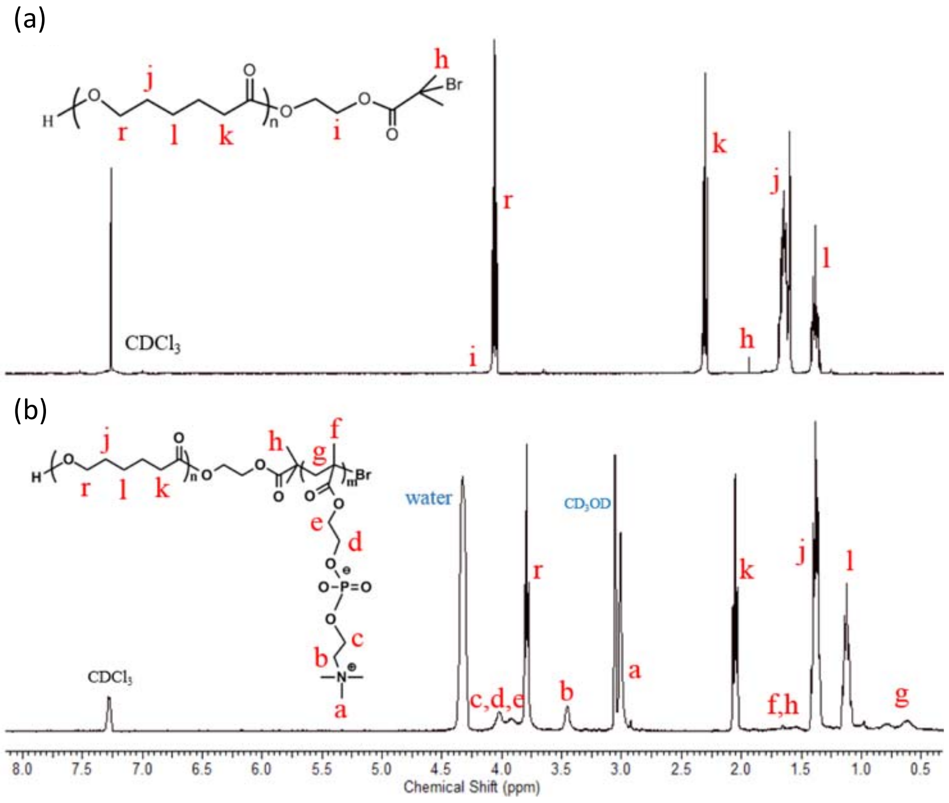

Figure S1 ^{1}H-NMR spectrum of (a) PCL-Br in $CDCl_3$ and (b) PCL-*b*-PMPC copolymers in $CDCl_3/CD_3OD$ (v/v, 1:1)

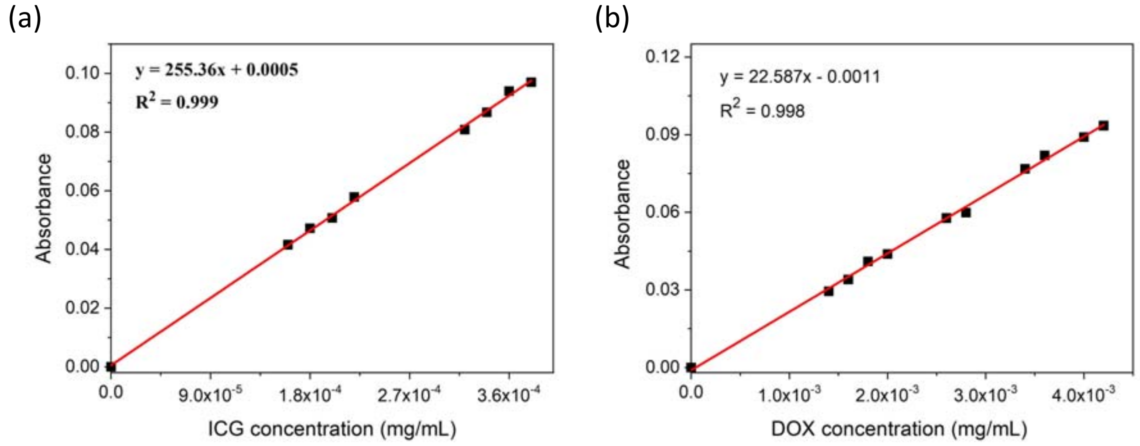

Figure S2 The calibration curves of (a) ICG and (b) DOX in $MeOH/CHCl_3$ (1:2, v/v) mixture.

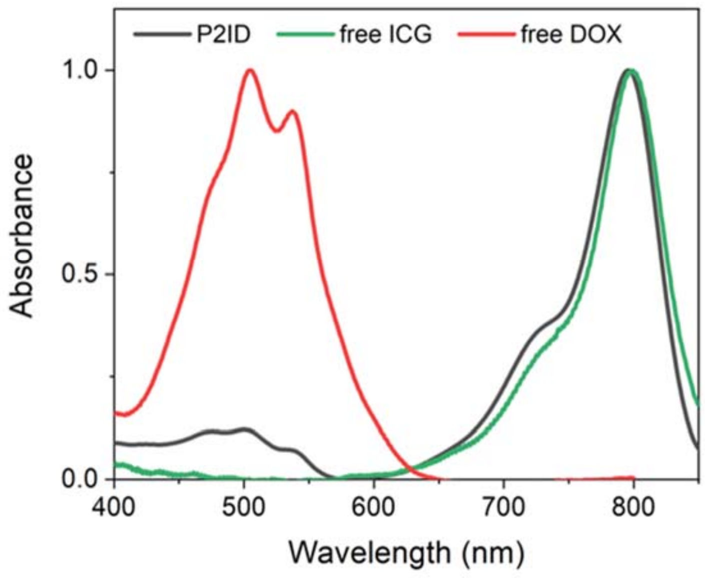

Figure S3 UV-vis spectrum of free ICG and free DOX (in solution, $CHCl_3$/MeOH, 2/1, v/v), and both DOX and ICG-loaded nanofibers (**P2ID**).

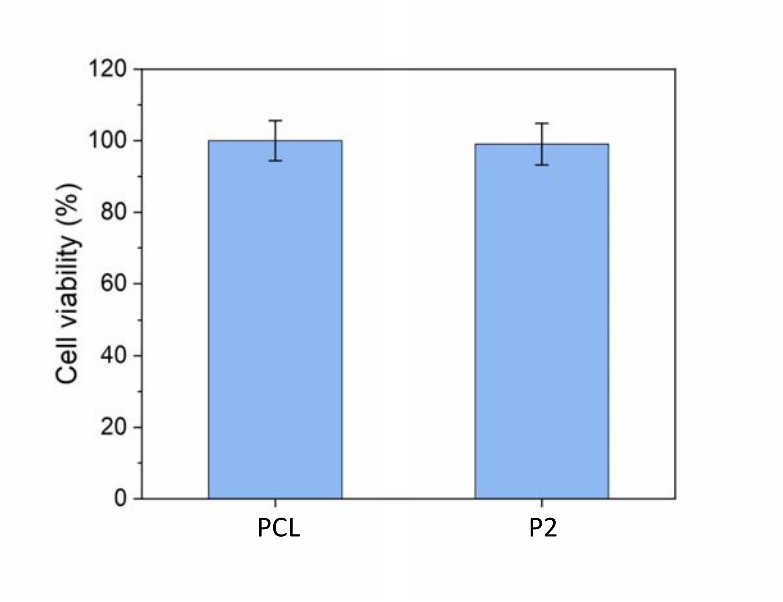

Figure S4 Cell viability of L929 cells treated with plain **PCL** and **P2** nanofibers.
